# Azithromycin Affords Neuroprotection in Rat Undergone Transient Focal Cerebral Ischemia

**DOI:** 10.3389/fnins.2019.01256

**Published:** 2019-11-26

**Authors:** Diana Amantea, Francesco Petrelli, Rosaria Greco, Cristina Tassorelli, Maria Tiziana Corasaniti, Paolo Tonin, Giacinto Bagetta

**Affiliations:** ^1^Section of Preclinical and Translational Pharmacology, Department of Pharmacy, Health and Nutritional Sciences, University of Calabria, Rende, Italy; ^2^Headache Science Center, IRCCS Mondino Foundation, Pavia, Italy; ^3^Department of Brain and Behavioral Sciences, University of Pavia, Pavia, Italy; ^4^Department of Health Sciences, University “Magna Graecia” of Catanzaro, Catanzaro, Italy; ^5^Regional Center for Serious Brain Injuries, S. Anna Institute, Crotone, Italy

**Keywords:** azithromycin, drug repurposing, ischemic stroke, neuroprotection, STAT3

## Abstract

Repurposing existing drugs represents a promising approach for successful development of acute stroke therapies. In this context, the macrolide antibiotic azithromycin has been shown to exert neuroprotection in mice due to its immunomodulatory properties. Here, we have demonstrated that acute administration of a single dose of azithromycin upon reperfusion produces a dose-dependent (ED_50_ = 1.40 mg/kg; 95% CI = 0.48–4.03) reduction of ischemic brain damage measured 22 h after transient (2 h) middle cerebral artery occlusion (MCAo) in adult male rats. Neuroprotection by azithromycin (150 mg/kg, i.p., upon reperfusion) was associated with a significant elevation of signal transducer and activator of transcription 3 (STAT3) phosphorylation in astrocytes and neurons of the peri-ischemic motor cortex as detected after 2 and 22 h of reperfusion. By contrast, in the core region of the striatum, drug administration resulted in a dramatic elevation of STAT3 phosphorylation only after 22 h of reperfusion, being the signal mainly ascribed to infiltrating leukocytes displaying an M2 phenotype. These early molecular events were associated with a long-lasting neuroprotection, since a single dose of azithromycin reduced brain infarct damage and neurological deficit measured up to 7 days of reperfusion. These data, together with the evidence that azithromycin was effective in a clinically relevant time-window (i.e., when administered after 4.5 h of MCAo), provide robust preclinical evidence to support the importance of developing azithromycin as an effective acute therapy for ischemic stroke.

## Introduction

Repurposing existing drugs for novel applications represents a promising strategy for the identification of effective therapies, especially in those clinical settings, such as ischemic stroke, where traditional drug discovery approaches have led to disappointing results (Ginsberg, 2008; Grupke et al., 2015). Due the pivotal role of the immune system in the complex cascade of events that contributes to the progression of ischemic brain injury, a number of preclinical studies have investigated the neuroprotective properties of drugs marketed for diverse indications, sharing relevant immunomodulatory properties (Amantea and Bagetta, 2016). In this context, the semisynthetic macrolide antibiotic azithromycin (9-deoxy-9a-aza-9a-methyl-9a-homoerythromycin A), approved worldwide for the treatment of various community-acquired infections, displays robust immunomodulatory and anti-inflammatory effects in chronic inflammatory airway diseases (Parnham et al., 2014; Cramer et al., 2017). The ability to modulate the immune system has been demonstrated to underlie the neuroprotective properties produced by a subchronic treatment with this macrolide antibiotic in adult mice subjected to spinal cord injury (Zhang et al., 2015; Gensel et al., 2017). More interestingly, we recently observed that a single dose of azithromycin was effective in ameliorating histological and functional outcomes in mice subjected to cerebral ischemia, by inhibiting brain infiltration of inflammatory myeloid cells and by promoting polarization of microglia/macrophages toward beneficial M2 phenotypes (Amantea et al., 2016a, b). Neuroprotection by azithromycin was prevented by inhibiting peripheral (i.e., in peritoneal macrophages) activity of arginase, an enzyme that mediates most M2-induced anti-inflammatory and immunosuppressive effects (Pesce et al., 2009; Murray and Wynn, 2011). Nevertheless, to date, the molecular mechanism(s) by which azithromycin promotes arginase activity and M2 polarization in immune cells has not been clarified. Given the crucial role of signal transducer and activator of transcription 3 (STAT3) in the induction of arginase 1 and, thus, in macrophage polarization toward the M2 phenotype (Vasquez-Dunddel et al., 2013; Gao et al., 2014; Liu et al., 2018; Zhao et al., 2018), we have hypothesized that this transcription factor may represent a downstream target of azithromycin. Thus, the main aims of this work consisted in (1) characterizing the neuroprotective properties of azithromycin in a second species, the rat, exposed to focal cerebral ischemia and (2) evaluating whether neuroprotection was associated with modulation of STAT3 phosphorylation in distinct cells populating the ischemic hemisphere. In particular, we aimed at characterizing the dose-response and therapeutic window for the neuroprotective effects of azithromycin in adult rat, using a single-dose acute administration, in order to validate the treatment schedule that would be feasible and could be implemented in stroke clinical trials.

## Materials and Methods

### Animals and Drug Treatments

Experiments were performed on adult male Wistar rats, weighing 280–320 g (Charles River, Calco, Como, Italy). Animals were housed under controlled environmental conditions with ambient temperature of 22°C, relative humidity of 65% and 12 h light:12 h dark cycle, with free access to food and water.

Animal care and experimental procedures were carried out following the guidelines of the Italian Ministry of Health (DM 116/1992 and DL 26/2014), in accordance with the European Union Directive 2010/63/EU. The protocols (numbers 120000344 and 1277/2015-PR) were approved by the Committee set by the Ministry of Health at the National Institute of Health (Rome). All efforts were made to reduce the number of animals used and their suffering and the study was conducted in compliance with the ARRIVE guidelines (Kilkenny et al., 2010).

Animals were randomly allocated to vehicle or drug treatments using the function = Rand() in Microsoft Excel to generate a randomized list that was then combined with the list corresponding to each experimental group. Azithromycin (Zithromax^®^, azithromycin dihydrate for injection, Pfizer) was dissolved in saline (0.9% NaCl) and administered at the indicated doses (0.15–150 mg/kg for the dose-response study, 150 mg/kg for Western blotting, immunofluorescence and time-window experiments) by the intraperitoneal (i.p.) route upon reperfusion (i.e., after 2 or 4.5 h of MCAo). Vehicle-control animals received an i.p. injection of saline (1 ml/kg).

An *a priori* power analysis was conducted to determine the minimal sample size needed to obtain a power of 80% at a significance level of 0.05 (OpenEpi software 3.01, Open Source Statistics for Public Health). Based on our previous experience with the MCAo model, we hypothesized a difference in ischemic volume between rats injected with vehicle and rats treated with azithromycin of about 100 mm^3^ (approximately 20% reduction of infarct size) and a variability (standard deviation) of 30. Thus, we estimated a sample size of at least 4 rats for each experimental group.

### Occlusion of the Middle Cerebral Artery

Focal brain ischemia was induced by proximal occlusion of the middle cerebral artery (MCAo), using a relatively non-invasive technique previously described (Amantea et al., 2010). Briefly, rats were anesthetized with 1.5–2% isoflurane vaporized in air and a silicone-coated nylon filament (diameter: 0.37 mm, Doccol Corporation, Redlands, CA, United States) was advanced through the external carotid artery into the internal carotid artery for approximately 18 mm from the common carotid artery bifurcation, up to the circle of Willis were a mild resistance and a 80–90% cortical cerebral blood flow (CBF, measured by lased-Doppler flowmetry; Periflux System 5000, Perimed, Sweden) reduction was indicative of successful vessel occlusion. Nine animals were excluded from the study because of unsuccessful MCAo. To allow reperfusion, the filament was withdrawn 2 or 4.5 h after MCAo. Despite all efforts were made to optimize surgical conditions and post-surgical care, six animals died during or immediately after surgery.

### Neuropathology and Assessment of Neurological Deficit

Twenty-four hours after the induction of ischemia, cerebral infarct volume was evaluated by the 2,3,5-triphenyltetrazolium chloride (TTC)-staining technique. Briefly, the brains were rapidly dissected and cut at 2-mm intervals from the frontal pole using a rat brain matrix (Harvard Apparatus, Massachusetts, United States) to obtain eight serial coronal sections that were stained in a TTC solution (2% in saline) at 37°C for 10 min.

To evaluate infarct volume 7 days after MCAo, the brains were dissected from the skull and immediately frozen. Using a cryostat, eight 20 μm-thick coronal sections were cut, at 2 mm intervals from the frontal pole, and stained with cresyl violet.

Images of TTC- or cresyl violet-stained sections were captured by a digital scanner and analyzed through an image analysis software (ImageJ, version 1.30). Infarct volume (expressed in mm^3^) was blindly determined by summing the infracted (pale) areas of the eight tissue slices and multiplying the obtained value by the interval-thickness between sections (2 mm). Infarct edema was calculated by subtracting the size of the whole contralateral (non-infarcted) hemisphere from the whole ipsilateral (infarcted) hemisphere.

Neurological examination was blindly performed every day, up to 7 days after MCAo, by scoring the deficits on a modified five-tiered grading system based on that developed by Longa et al. (1989) as follows: 0, no deficit; 1, failure to fully extend the contralateral forepaw when held by the tail; 2, reduced resistance to a lateral push; 3, turning toward the contralateral side when held by the tail on a flat surface; 4, falling to contralateral side; 5, no spontaneous movement with depressed level of consciousness.

### Western Blot Analysis

Individual tissue samples were obtained at the level of the MCA territory (1.7 to −3.3 mm from Bregma) by rapidly dissecting the areas corresponding to the ipsilateral (ischemic) and contralateral motor cortex and striatum of vehicle- or azithromycin-injected rats subjected to MCAo followed by 2 or 22 h of reperfusion (*n* = 4 animals per group). Tissue pieces were then homogenized in a glass homogenizer using six volumes of ice-cold lysis buffer containing 50 mM Tris–HCl, pH 7.5, 150 mM NaCl, 2 mM EDTA, 2 mM EGTA, 1% Triton, 1 nM okadaic acid, a cocktail of protease inhibitors (code P8340, Sigma, Milan, Italy) and a cocktail of phosphatase inhibitors (code 524625, Calbiochem, La Jolla, CA, United States). Samples were then centrifuged at 10,000 × *g* for 15 min at 4°C and protein concentration in the supernatant was determined by the DC protein assay (Bio-Rad Laboratories, Milan, Italy).

Proteins were resolved by sodium dodecyl sulphate-polyacrylamide gel electrophoresis (SDS-PAGE) and electrotransferred to nitrocellulose membranes (Optitran BA-S 83, Schleicher & Schuell Bioscence, Dassel, Germany). Blots were probed overnight at 4°C with the following primary antibodies: a rabbit polyclonal antibody for phosphorylated (Tyr705) STAT3 (p-STAT3) at 1:1000 dilution (code 9131, Cell Signaling Technology, Danvers, MA, United States), a rabbit polyclonal antibody for STAT3 at 1:1000 dilution (code 9132, Cell Signaling Technology), a mouse monoclonal anti-alpha tubulin antibody at 1:2000 dilution (acetyl K40; Abcam, Cambridge, MA, United States). Following incubation with the corresponding horseradish peroxidase-conjugated secondary antibodies for 1 h at room temperature, immunoreactivity was visualized by enhanced chemiluminescent detection (Amersham Biosciences, GE Healthcare, Milan, Italy) and exposure to X-ray films (Hyperfilm ECL, Amersham Biosciences). After scanning the autoradiographic films, the obtained digital images were subjected to densitometric analysis using ImageJ 1.50b (National Institute of Health, United States).

### Immunohistochemistry

After 2 and 22 h of reperfusion, animals were anesthetized with isoflurane (5% in air) and perfused through the heart with saline (0.9% NaCl) followed by 4% paraformaldehyde in phosphate buffer (PB; 0.1 M; pH 7.4), containing 50 mM NaF. Each brain was rapidly removed, post-fixed in the same fixative for 2 h and cryoprotected in 30% sucrose solution in PB at 4°C. 40 μm-thick coronal brain sections, at the level of the MCA territory (1.7 to −3.3 mm from Bregma), were obtained using a cryostat and collected in PB.

For p-STAT immunostaining, tissue slices were pre-incubated with 1% NaOH and 1% H_2_O_2_ in H_2_O for 20 min, 0.3% glycine (in PB) for 10 min, and 0.03% sodium dodecyl sulfate (in PB) for 10 min. After two washes in PB and a pre-incubation for 1 h in blocking solution (3% normal donkey serum, 0.2% Triton X-100, 0.2% sodium azide in PB), the primary antibodies were added to the incubation medium for 24 h at 4°C. Colocalization studies were performed using a combination of the following primary antibodies: rabbit polyclonal anti-p-STAT3 (1:200 dilution; Tyr705, Cell Signaling Technology), mouse anti-GFAP (anti-glial fibrillary acidic protein; 1:200 dilution; code AB5804, Merck Millipore, Milan, Italy) to label astrocytes, or mouse anti-NeuN (anti-neuronal nuclei; 1:200 dilution; MAB377, Chemicon International, Temecula, CA, United States) to label neurons, mouse anti-CD11b (clone OX-42; 1:200 dilution; code MCA275, Bio-Rad Laboratories, Milan, Italy) to label myeloid cells, rabbit anti-Iba-1 (ionized calcium-binding adaptor molecule-1; 1:500; code 019-19741, Wako Pure Chemicals, Japan) to label microglia/macrophages, goat anti-arginase I (N-20; 1:100; code 18351, Santa Cruz Biotechnology, Germany) and rabbit anti-Ym1 (1:100; code 60130, StemCell Technologies, United Kingdom) to label alternatively activated microglia/macrophages.

Thereafter, sections were incubated for 2 h at room temperature, in the dark, in a solution containing an appropriate mixture of the corresponding secondary antibodies labeled with Alexa Fluor 488 or Alexa Fluor 594 (1:400 dilution; Molecular Probes, Invitrogen, Milan, Italy). Finally, nuclei were counterstained with 4′,6-diamidino-2-phenylindole (DAPI, 1:500; Sigma-Aldrich, Milan, Italy), and the sections were mounted on positively charged slides and coverslipped with Fluoromount (Diagnostic BioSystems, Pleasanton, CA, United States). Immunostaining was observed using a fluorescence microscope (Leica DMI6000B) equipped with a high-resolution digital camera (Leica DFC350FX) and a dedicated software (LAS AF6000) for image analysis and deconvolution.

### Statistical Analysis

Data are expressed as mean ± standard error of the mean (SEM) and subjected to statistical analysis (repeated measures or two-way ANOVA followed by Bonferroni multiple comparison test) using Graph-Pad Prism version 5.00 for Windows (GraphPad Software, San Diego, CA, United States). Statistical significance was accepted at the 95% confidence level (*P* < 0.05).

## Results

**FIGURE 1:**
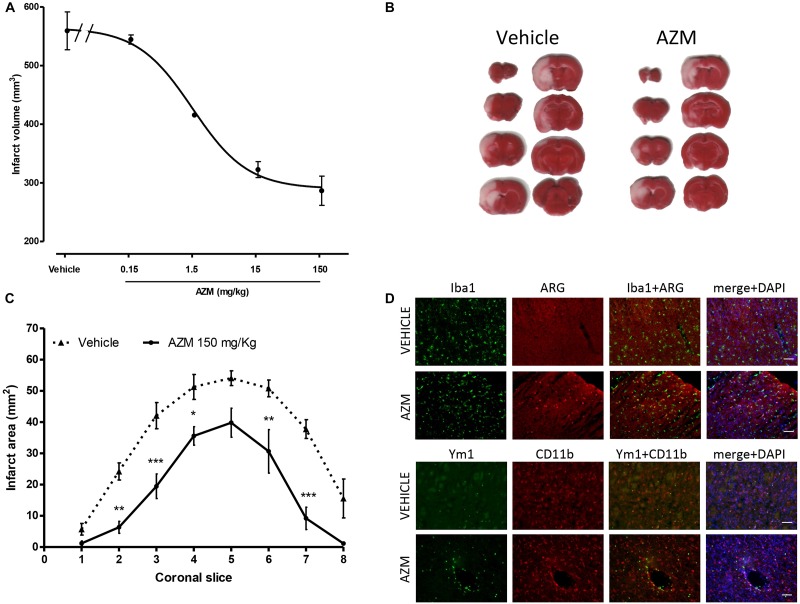
Neuroprotective properties of azithromycin in rats subjected to 2 h MCAo followed by 22 h of reperfusion. **(A)** Intraperitoneal administration of a single dose of azithromycin (AZM, 0.15–150 mg/kg) upon reperfusion results in a dose-dependent (ED_50_ = 1.40 mg/kg; 95% CI = 0.48–4.03) reduction of ischemic brain damage measured after 22 h of reperfusion (*n* = 4–6 animals per experimental group). **(B)** Representative TTC-stained coronal brain sections and **(C)** corresponding values of ischemic areas in rats subjected to transient MCAo and injected i.p. with vehicle (0.9% NaCl) or AZM (150 mg/kg) upon reperfusion (^∗^*P* < 0.05, ^∗∗^*P* < 0.01, and ^∗∗∗^*P* < 0.001 vs. corresponding vehicle, ANOVA for repeated measures followed by Bonferroni post-test). **(D)** Representative immunofluorescence images of the ischemic frontoparietal cortex (upper set of panels) and striatum (lower set of panels) of rats injected with vehicle (0.9% NaCl) or AZM (150 mg/kg) i.p. upon reperfusion. Brain tissue slices were stained for Iba1 (to label microglia/macrophages) or CD11b (to label microglia/macrophages and neutrophils) and arginase 1 (ARG) or Ym1 (markers of the M2 phenotype), whereas, nuclei were counterstained with DAPI.

The first objective of the present study consisted in characterizing the neuroprotective properties of azithromycin in rats subjected to transient MCAo. Systemic, i.p., administration of azithromycin upon reperfusion (i.e., after 2 h of MCAo) resulted in a dose-dependent (ED_50_ = 1.40 mg/kg; 95% CI = 0.48–4.03) reduction of ischemic brain damage measured 24 h after the initial insult (Figure 1A). As shown in representative TTC-stained sections (Figure 1B), the drug (150 mg/kg, i.p.) reduced infarct extent in peri-ischemic regions of the motor and frontal cortex, and of the ventral striatum, thus involving the entire hemisphere (Figure 1C). At this dose, azithromycin also significantly reduced cerebral edema produced by 2h MCAo followed by 22 h of reperfusion (edema: vehicle 85.7 ± 19.9 mm^3^; AZM 25.2 ± 9.6 mm^3^, *P* < 0.05, *t*-test, *n* = 6). In agreement with our previous observation in mice (Amantea et al., 2016b), the neuroprotective effects of azithromycin were not associated with blood flow modifications, since the reduction of CBF induced by MCAo and the re-establishment of cerebral perfusion after intraluminal filament withdrawal were not influenced by drug administration (data not shown). Moreover, similarly to mice, neuroprotection by azithromycin was associated with elevation of the M2 markers arginase 1 and Ym1, both in the cortex and in the striatum, being in the latter area the Ym1 signal abundant in CD11b immunopositive cells, likely resembling monocytes and neutrophils infiltrating from blood vessels (Figure 1D).

**FIGURE 2:**
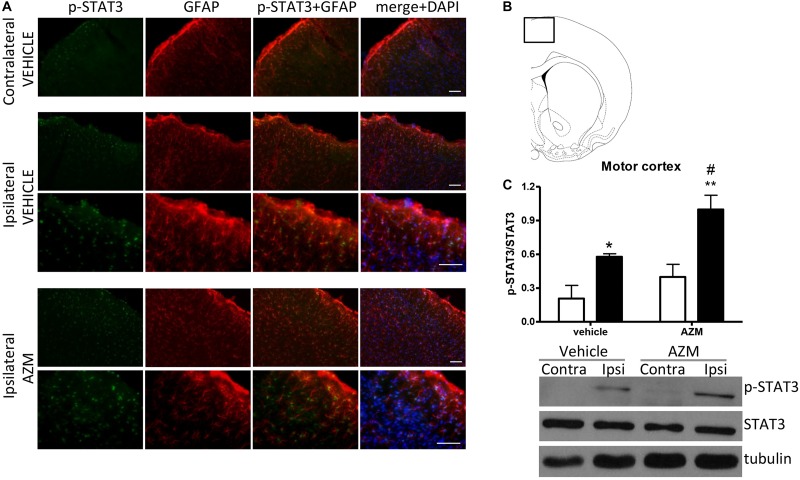
Azithromycin elevates STAT3 phosphorylation in the motor cortex of rats subjected to 2 h MCAo followed by 2 h of reperfusion. **(A)** Representative immunofluorescence images of the contralateral and ipsilateral (ischemic) motor cortex (inset in **B**) of rats subjected to transient MCAo, showing expression of p-STAT3 in most GFAP-positive astrocytes and other DAPI-positive cells (likely resembling pyramidal neurons) in the lesioned hemisphere. Scale bars = 70 μm. **(C)** Western blot analysis of phospho-STAT3 (Tyr705) (p-STAT3), total STAT3, and tubulin performed on brain homogenates from the ipsilateral (Ipsi, dark bars) and contralateral (Contra, white bars) motor cortex. Rats were treated with vehicle (0.9% NaCl) or azithromycin (AZM, 150 mg/kg), i.p., upon reperfusion. Data from the densitometric analysis of the autoradiographic bands are expressed as mean ± SEM of four independent experiments (*n* = 4 rats per experimental group); ^∗^*P* < 0.05 vs. vehicle contralateral; ^#^*P* < 0.05 vs. AZM contralateral and ^∗∗^*P* < 0.01 vs. vehicle ipsilateral (two-way ANOVA for repeated measures followed by Bonferroni post-test).

**FIGURE 3:**
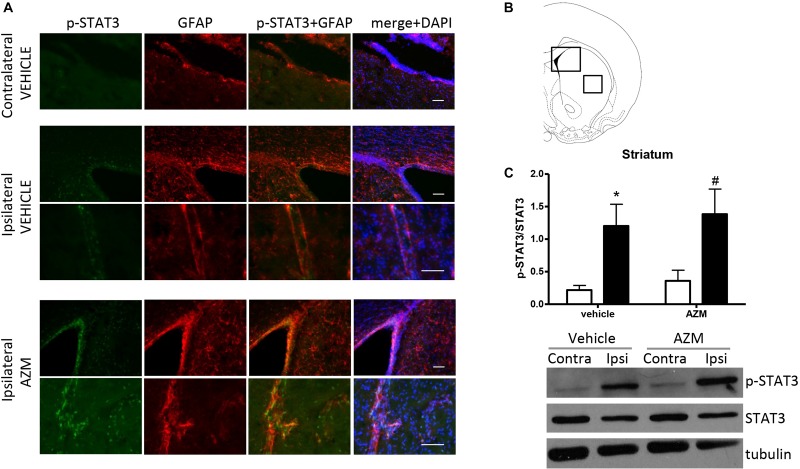
Azithromycin does not affect elevation of STAT3 phosphorylation observed in the ischemic striatum of rats subjected to 2 h MCAo followed by 2 h of reperfusion. **(A)** Representative immunofluorescence images of the contralateral and ipsilateral (ischemic) striatum (insets in **B**) of rats subjected to transient MCAo showing expression of p-STAT3 in some GFAP-positive periventricular astrocytes and in ependymal cells lining the lateral ventricle. Expression of p-STAT3 is also evident in intravascular leukocytes recruited to the ipsilateral striatum of both vehicle-injected and azithromycin-treated animals. Scale bars = 70 μm. **(C)** Western blot analysis of phospho-STAT3 (Tyr705) (p-STAT3), total STAT3 and tubulin performed on brain homogenates from the ipsilateral (Ipsi, dark bars) and contralateral (Contra, white bars) striatum. Rats were treated with vehicle (0.9% NaCl) or azithromycin (AZM, 150 mg/kg), i.p., upon reperfusion. Data from the densitometric analysis of the autoradiographic bands are expressed as mean ± SEM of four independent experiments (*n* = 4 rats per experimental group); ^∗^*P* < 0.05 vs. vehicle contralateral and ^#^*P* < 0.05 vs. AZM contralateral (two-way ANOVA for repeated measures followed by Bonferroni post-test).

In order to investigate the molecular pathways implicated in the effect of azithromycin, we have measured the level of phosphorylation of STAT3 in the motor cortex and the striatum of rats subjected to transient MCAo. By western blotting analysis, we detected a significant elevation of STAT3 phosphorylation in the ischemic cortex of vehicle-injected rats as compared to the corresponding contralateral tissue (Figure 2C). Expression of p-STAT3 mainly occurred in the nuclei of GFAP-positive astrocytes activated in this brain region following the ischemic insult (Figure 2A). Interestingly, acute administration of azithromycin (150 mg/kg, i.p. upon reperfusion) further elevated phosphorylation of STAT3 in this peri-infarct region (Figure 2C). Immunofluorescence analysis showed that increased p-STAT3 not only occurred in GFAP-positive astrocytes, but also in other DAPI-positive cells, likely resembling cortical pyramidal neurons (Figure 2A). At this early reperfusion time, STAT3 phosphorylation also resulted to be elevated in the ipsilateral striatum of vehicle-injected and azithromycin-treated rats (Figure 3). In this brain region, the effect of drug treatment did not differ from control-vehicle (Figure 3C), and immunofluorescence analysis revealed that p-STAT3 was mainly expressed in ependymal cells lining the lateral ventricle and in some GFAP-immunopositive periventricular astrocytes (Figure 3A). Moreover, pSTAT3 was detected in intravascular leukocytes of vehicle-injected rats, showing a more intense signal in azithromycin-treated animals (Figure 3A).

**FIGURE 4:**
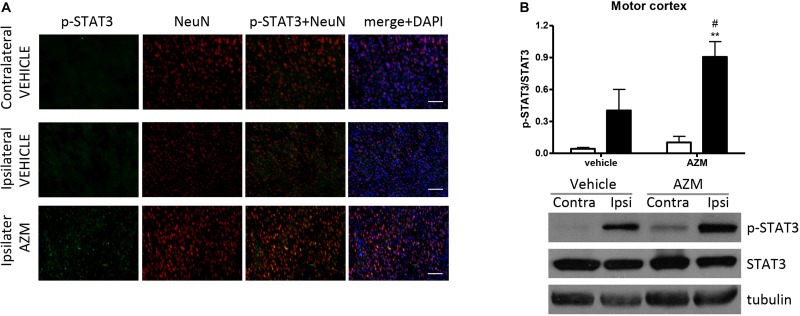
Azithromycin elevates STAT3 phosphorylation in the motor cortex of rats subjected to 2 h MCAo followed by 22 h of reperfusion. **(A)** Representative immunofluorescence images of the contralateral and ipsilateral (ischemic) motor cortex of rats subjected to transient MCAo showing expression of p-STAT3 mainly in NeuN-positive neurons. Scale bars = 70 μm. **(B)** Western blot analysis of phospho-STAT3 (Tyr705) (p-STAT3), total STAT3, and tubulin performed on brain homogenates from the ipsilateral (Ipsi, dark bars) and contralateral (Contra, white bars) motor cortex. Rats were treated with vehicle (0.9% NaCl) or azithromycin (AZM, 150 mg/kg), i.p., upon reperfusion. Data from the densitometric analysis of the autoradiographic bands are expressed as mean ± SEM of four independent experiments (*n* = 4 rats per experimental group); ^∗∗^*P* < 0.01 vs. AZM contralateral and ^#^*P* < 0.05 vs. vehicle ipsilateral (two-way ANOVA for repeated measures followed by Bonferroni post-test).

**FIGURE 5:**
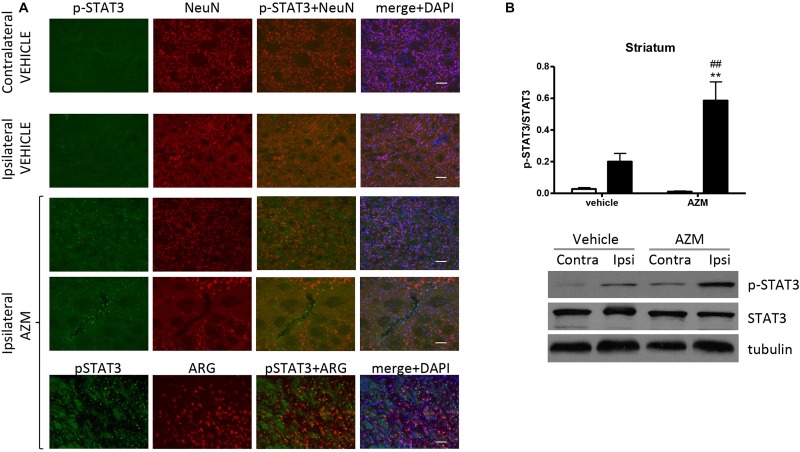
Azithromycin elevates STAT3 phosphorylation in the ischemic striatum of rats subjected to 2 h MCAo followed by 22 h of reperfusion. **(A)** Representative immunofluorescence images of the contralateral and ipsilateral (ischemic) striatum of rats subjected to transient MCAo showing expression of p-STAT3 in some NeuN-positive neurons of the ischemic hemisphere. An increased number of p-STAT3 immunopositive cells can be observed in the ispilateral striatum of rats treated with azithromycin (AZM), being the signal mainly coincident with infiltrating leukocytes and arginase 1 (ARG)-immunopositive cells (lower panels), namely microglia/macrophages displaying an M2 phenotype. Scale bars = 70 μm. **(B)** Western blot analysis of phospho-STAT3 (Tyr705) (p-STAT3), total STAT3 and tubulin performed on brain homogenates from the ipsilateral (Ipsi, dark bars) and contralateral (Contra, white bars) striatum. Rats were treated with vehicle (0.9% NaCl) or azithromycin (AZM, 150 mg/kg), i.p., upon reperfusion. Data from the densitometric analysis of the autoradiographic bands are expressed as mean ± SEM of four independent experiments (*n* = 4 rats per experimental group); ^∗∗^*P* < 0.01 vs. vehicle ipsilateral and ^##^*P* < 0.01 vs. AZM contralateral (two-way ANOVA for repeated measures followed by Bonferroni post-test).

After 22 h of reperfusion, systemic treatment with azithromycin resulted in elevated STAT3 phosphorylation in the ipsilateral motor cortex as compared with corresponding tissue from vehicle-injected rats (Figure 4). The immunofluorescence signal corresponding to p-STAT3 co-localized mainly with NeuN-positive neurons (Figure 4A) and, to a lesser extent, with astrocytes (data not shown). By contrast, in the core region of the striatum, neuronal expression of p-STAT3 was only rarely observed, since this transcription factor was mainly present in leukocytes infiltrating from blood vessels (Figure 5A). Interestingly, the signal corresponding to p-STAT3 was detected in numerous cells expressing arginase 1, thus resembling an alternatively activated M2 phenotype (Figure 5A, lower panels) that were abundant in the ischemic hemisphere of azithromycin-treated animals (Figure 1D). Accordingly, western blotting analysis showed that treatment with azithromycin (150 mg/kg, i.p., upon reperfusion) significantly elevated levels of STAT3 phosphorylation in the striatum after 22 h of reperfusion (Figure 5B).

**FIGURE 6:**
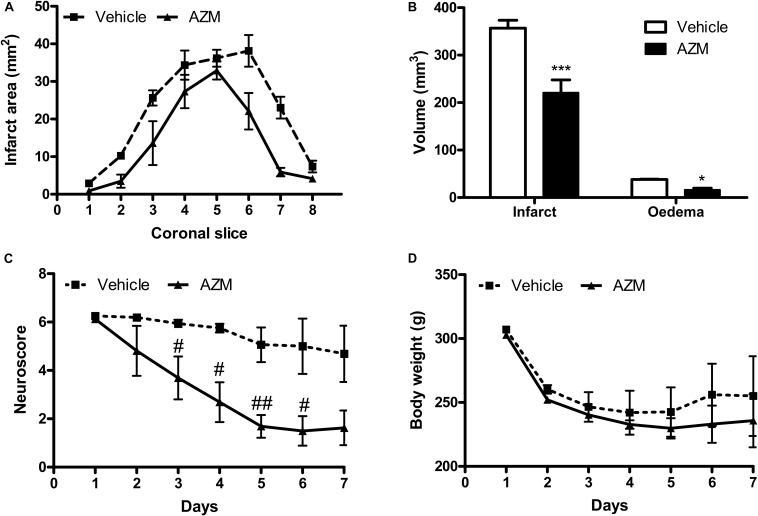
Systemic administration of a single dose of azithromycin exerts neuroprotection up to 7 days after reperfusion in rats subjected to 2 h MCAo. **(A)** Ischemic area, **(B)** infarct and edema volumes, **(C)** neurological deficit and **(D)** body weight of rats subjected to 2 h MCAo followed by 7 days of reperfusion. Rats were injected i.p. with vehicle (0.9% NaCl) or azithromycin (AZM, 150 mg/kg) upon reperfusion. Data are expressed as mean ± SEM of four independent experiments (*n* = 4 rats per experimental group); ^∗∗∗^*P* < 0.001 and ^∗^*P* < 0.05 vs. corresponding vehicle (two-way ANOVA followed by Bonferroni *post hoc* test); ^#^*P* < 0.05 and ^##^*P* < 0.01 vs. corresponding vehicle (ANOVA for repeated measures followed by Bonferroni post-test).

In order to assess whether the early molecular effects detected would result in long-lasting neuroprotection, we have measured the effects of a single dose of azithromycin (150 mg/kg, i.p., upon reperfusion) on brain infarct damage and neurological deficit produced by 2 h MCAo. As shown in Figure 6, drug administration resulted in significant reduction of cerebral infarct and edema volumes, as well as in amelioration of neurological deficit caused by transient ischemia. Administration of a single lower dose (i.e., 15 mg/kg) did not affect MCAo-associated delayed behavioral impairment (data not shown). In the animals treated with azithromycin, no manifest adverse effects were observed, neither the drug affected body weight or mortality that were comparable to those observed in vehicle-injected group.

**FIGURE 7:**
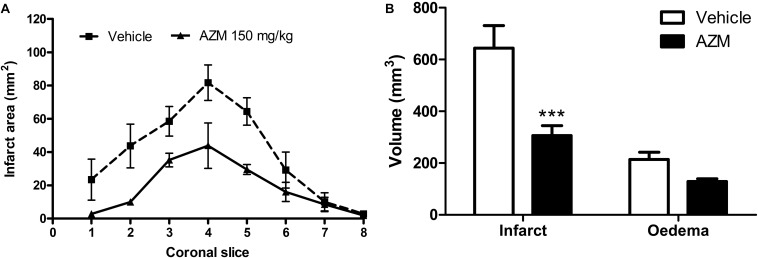
A single dose of azithromycin administered 4.5 h after MCAo reduces ischemic brain damage measured 24 h after the insult. **(A)** Infarct area and **(B)** volumes of infarct and edema of rats subjected to 4.5 h of MCAo followed by 19.5 h of reperfusion. Rats were injected i.p. with vehicle (0.9% NaCl) or azithromycin (AZM, 150 mg/kg) upon reperfusion. Data are expressed as mean ± SEM of four independent experiments (*n* = 4 rats per experimental group); ^∗∗∗^*P* < 0.001 vs. vehicle (two-way ANOVA followed by Bonferroni *post hoc* test).

Finally, to further strengthen the translational value of our findings, we have administered azithromycin upon reperfusion in rats subjected to 4.5 h of MCAo, namely resembling the latest time-window at which pharmacological thrombolysis is currently approved. Under these experimental conditions, that produced a more sever ischemic damage in rats as compared with 2 h MCAo (Figure 1), we observed a significant reduction of brain infarct volume in the animals treated with azithromycin (150 mg/kg, i.p.) upon reperfusion (Figure 7).

## Discussion

The results of the present work demonstrate that administration of a single dose of azithromycin reduces brain infarct damage and ameliorates neurological deficit produced by transient focal cerebral ischemia in adult rats. The ED50 (1.40 mg/kg) is lower than the bactericidal dose of the drug in rodents and is approximately 50 times lower than the intravenous dose demonstrated to be well tolerated in humans (Luke et al., 1996). The neuroprotective properties of this macrolide antibiotic occur within an extended time-window, since the drug was effective when administered up to 4.5 h after MCAo and neuroprotection was maintained up to 7 days after the insult. Neuroprotection was associated with elevation of STAT3 phosphorylation in distinct cells of the ischemic brain, such as neurons, astrocytes and innate immune cells, also including those infiltrating from the periphery displaying an M2 phenotype. This latter evidence is consistent with previous findings (Amantea et al., 2016a, b) and further supports the concept that the immunomodulatory properties of azithromycin underlie its beneficial effects in ischemic conditions. Taken together, the present data extend our previous observations in mice (Amantea et al., 2016a, b; Petrelli et al., 2016), thus providing robust preclinical evidence to encourage the development of azithromycin as an effective acute therapy for ischemic stroke.

In fact, as suggested by widely accepted recommendations for effective translational research in stroke, confirmation of efficacy in at least a second species is expected to enhance the chance of success in large-scale clinical trials (Fisher et al., 2009; Lapchak et al., 2013). Moreover, azithromycin fulfils most prerequisites proposed in these preclinical guidelines for an ideal neuroprotective drug, namely the ability to improve short- and long-term histological outcomes in randomized and blinded studies, consideration of a clinically useful therapeutic window and dose, demonstration of the efficacy of a clinically relevant route of administration (Amantea et al., 2016a) and efficacy demonstrated in more than two laboratories that use different models and strains (Petrelli et al., 2016; Varano et al., 2017; Barks et al., 2019). In a neonatal rat model of hypoxic-ischemic cerebral injury, either acute or chronic post-treatment with azithromycin reduced sensorimotor deficits and brain damage severity (Barks et al., 2019). In that study, the neuroprotection was lost if drug administration (45 mg/kg) was delayed further than 2 h after the insult (Barks et al., 2019). The apparent discrepancy with our findings, showing that a single dose of azithromycin is effective if administered up to 4.5 h after the insult, may stem in the higher dose we tested (150 mg/kg) and in the different experimental model used. Moreover, Barks et al. (2019) demonstrated that administration of a single dose (i.e., 40 mg/kg, i.p.) of azithromycin yielded blood levels similar to human regimens, whereby brain concentrations persisted for several days after injection and long after blood levels were declined, further supporting previous evidence documenting that azithromycin effectively crosses the blood-brain barrier and accumulates in cerebral tissue (Davila et al., 1991; Jaruratanasirikul et al., 1996). It is important to highlight that azithromycin is commonly used to treat infections in stroke patients and its safety in these patients has already been established, at least when administered several hours after the insult. In the present study, assessment of safety outcomes was limited to body temperature, body weight and survival: none of these parameters was affected by treatment with azithromycin. Moreover, in a recent retrospective trial, Smith et al. (2019) analyzed data from 2708 patients with ischemic stroke and with an infection, treated with systemic antibiotic therapy, grouped into eight different classes, during the first 2 weeks after stroke onset. The results of this study showed that treatment with macrolides was independently associated with a favorable clinical outcome in any infection or pneumonia complicating stroke (Smith et al., 2019). This was ascribed to the favorable immune-modulatory effects and, eventually, to the neuroprotective properties that macrolides display in preclinical stroke settings.

The progression of ischemic brain injury is dramatically influenced by the activation of innate immune cells, including local microglia and blood-borne monocyte-derived macrophages and neutrophils. These cells play a dualistic role as they may display pathological properties and contribute to the initial tissue injury, but have also the potential to adopt protective phenotypes that prompt anti-inflammatory effects and tissue repair (Iadecola and Anrather, 2011; Perego et al., 2016; García-Culebras et al., 2018). Accordingly, growing evidence supports the idea that promoting polarization toward protective M2 or N2 phenotypes ameliorates stroke outcomes in animal models (Cuartero et al., 2013; Certo et al., 2015; Amantea et al., 2018). By altering the phenotype of microglia and monocytes/macrophages, namely increasing M2-to-M1 ratio, azithromycin has been shown to improve recovery of mice subjected to transient MCAo (Amantea et al., 2016a, b) or spinal cord injury (Zhang et al., 2015; Gensel et al., 2017). Replication of the immunomodulatory effects of azithromycin in another species, namely the rat, is confirmatory of its neuroprotective efficacy. In fact, targets and mechanisms are not always the same across different species. A recent study provides proof-of-concept that species differences (rat vs. mouse vs. human) exist in chemokine/cytokine subnetworks in brain cells exposed to oxygen-glucose deprivation that may be relevant to stroke pathophysiology (Du et al., 2017). Rat vs. mouse differences in inflammatory gene induction after focal brain ischemia have also been found in whole brain tissue (Schroeter et al., 2003; Zhou et al., 2016). Thus, although rodent models may not be completely predictive for human stroke, especially concerning the validation of immunomodulatory drugs (Sharp and Jickling, 2014), reproducibility of the results in more than one species significantly strengthens the translational value of preclinical evidence.

A major issue that is still unresolved consists in the elucidation of the molecular mechanism(s) involved in the immunomodulatory effects of azithromycin. *In vitro* evidence demonstrates that azithromycin accumulates in macrophage lysosomes, where it increases pH, interacts with membrane lipids, triggers phospholipidosis and alters vesicular trafficking, thus impairing processes such as endocytosis and phagocytosis (Munić et al., 2011; Nujić et al., 2012a). Moreover, impairment of lysosomal functions by azithromycin also deregulates Toll-like receptor-4 recycling and signaling leading to anti-inflammatory phenotypes in LPS-stimulated J774A.1 cells (Nujić et al., 2012a). However, previous studies strongly suggest that the effects of azithromycin are due to a direct interaction with immune cells since the drug elevates the expression of a number of typical M2 genes and proteins, while it decreases M1-like phenotypic markers in cultured macrophages (Murphy et al., 2008; Vrančić et al., 2012; Choi et al., 2014; Zhang et al., 2015; Gensel et al., 2017). Interestingly, non-antibiotic analogs of azithromycin show similar anti-inflammatory effects both *in vitro* and *in vivo* (Mencarelli et al., 2011; Balloy et al., 2014). In this context, we have recently demonstrated that the ability to trigger transition toward M2 phenotypes in cerebral and peripheral innate immune cells (namely, microglia and monocytes/macrophages) underlies neuroprotection by an acute post-treatment with azithromycin in mice subjected to transient MCAo (Amantea et al., 2016b). In particular, we observed that neuroprotection by azithromycin could be prevented by inhibiting peripheral (i.e., in peritoneal macrophages) arginase activity that, in turn, mediates most M2-induced anti-inflammatory and immunosuppressive effects (Pesce et al., 2009; Murray and Wynn, 2011).

Interestingly, the arginase 1 promoter region displays multiple STAT3-binding elements, while phosphorylated STAT3 binds to multiple sites in the arginase 1 promoter (Vasquez-Dunddel et al., 2013). Accordingly, STAT3 signaling has been implicated in the induction of arginase 1 and, thus, in macrophage polarization toward the M2 phenotype (Vasquez-Dunddel et al., 2013; Gao et al., 2014; Liu et al., 2018; Zhao et al., 2018). Moreover, a pivotal mechanism triggering anti-inflammatory responses in myeloid cells involves IL-10/STAT3 signaling pathway (Takeda et al., 1999; Williams et al., 2004; Hutchins et al., 2012; Murray and Smale, 2012). Indeed, STAT3 has been shown to mediate IL-10-induced transition of murine macrophages toward protective phenotypes by inducing downstream anti-inflammatory genes, including arginase 1 and Ym1 (Lang et al., 2002; Koscsó et al., 2013; Barrett et al., 2017). Here, we demonstrate that elevation of arginase 1 and Ym1 expression produced by azithromycin is associated with increased phosphorylation of STAT3 in myeloid cells activated locally or recruited from the periphery. Notably, STAT3 is a pivotal mediator of the anti-inflammatory effects of IL-10 also in human macrophages (Williams et al., 2004) and is among the highly predicted target genes for the dysregulated miRNAs occurring in the peripheral blood mononuclear cells (PBMCs) of ischemic stroke patients (Bam et al., 2018). This further strengthens the translational value of our findings.

STAT3 is a ubiquitously expressed transcription factor that normally exerts a variety of (often opposite) functions (Hutchins et al., 2013). For instance, in addition to the above mentioned IL-10-stimulated M2 polarization, STAT3 activation may also trigger M1 polarization (Qin et al., 2012) and has been associated with increased pro-inflammatory responses in reactive astrocytes (Liu et al., 2019). Accordingly, previous work has demonstrated that elevation of p-STAT3 in the ischemic hemisphere may contribute to tissue damage (Satriotomo et al., 2006). This is consistent with the early (i.e., after 2 h of reperfusion) increase of STAT3 phosphorylation that we observe in the ischemic hemisphere of vehicle-injected animals, an effect that can thereafter be potentiated by azithromycin treatment through a derangement of the final response, from detrimental to beneficial. Indeed, previous studies have documented that STAT3 activation occurs in neurons, astrocytes and microglia/macrophages after focal cerebral infarction (Justicia et al., 2000; Suzuki et al., 2001; Amantea et al., 2011) and may provide beneficial effects through a variety of mechanisms (Jung et al., 2011; Gertz et al., 2012; Hoffmann et al., 2015). In neurons, STAT3 has been shown to contribute to neuronal survival and regeneration after diverse types of injury (Bareyre et al., 2011; Di Domenico et al., 2012; Niemi et al., 2016). In astrocytes, phosphorylation and nuclear translocation of STAT3 is required for their activation and their involvement in functional recovery after nervous system injury, including ischemia (Herrmann et al., 2008; Ceyzériat et al., 2016; Diaz et al., 2017). Thus, the elevation of STAT3 phosphorylation that we observe in neurons and astrocytes of the cortical penumbra is suggestive of a role of this factor in late tissue recovery promoted by azithromycin administration. This is further supported by the evidence that other substances that elevate STAT3 phosphorylation in neurons and astrocytes have been shown to exert neuroprotection in stroke models (Amantea et al., 2011; Hou et al., 2018; Chen et al., 2019).

While STAT3 phosphorylation may represent an important mechanism, the upstream molecular target on which azithromycin acts remains unclear. Using chemical proteomics approaches, based on compound-immobilized affinity chromatography, Nujić et al. (2012b) identified valosin containing protein (VCP) as a potential target of azithromycin in macrophages. Some VCP functions are crucial for cell survival (Bartolome et al., 2013; Ikeda et al., 2015), whereby VCP modulators have been shown to exert neuroprotection in models of ischemic retinal injury by suppressing intracellular ATP depletion and endoplasmic reticulum stress (Hata et al., 2017). Nevertheless, the effects of azithromycin on cytokines production in J774A.1 murine macrophages were not VCP dependent and the drug did not affect nuclear factor κB signaling in these cells (Nujić et al., 2012b). Although the authors excluded the contribution of VCP to the anti-inflammatory effects of azithromycin, it should be noted that VCP over-expression induces STAT3 phosphorylation in colorectal cancer cell lines (Fu et al., 2016). This highlights that VCP may represent an upstream target by which azithromycin elevates STAT3 phosphorylation in neurons and immune cells, but further work is necessary to demonstrate this intriguing, though very preliminary, hypothesis. The discovery of the upstream molecular target will be crucial to identify structural analogs that selectively mimic the neuroprotective and/or immunomodulatory properties of azithromycin without exerting antibacterial effects, thus reducing the risk of inadvertently prompt antibiotic resistance, dysbiosis and their consequences (Parnham et al., 2014; Blaser, 2016; Mahana et al., 2016). Indeed, various macrolide derivatives without antimicrobial activity have been shown to retain immunomodulatory properties in models of lung inflammation, inflammatory bowel diseases, arthritis, and skin inflammation (Mencarelli et al., 2011; Bosnar et al., 2012; Rodriguez-Cerdeira et al., 2012; Sugawara et al., 2012; Balloy et al., 2014; Hodge et al., 2017), whereas the ability of non-antibiotic derivatives to reduce macrophage−mediated neurotoxicity has recently been demonstrated *in vitro* (Zhang et al., 2019). However, it is pivotal to highlight that, at variance with chronic inflammatory conditions, for the acute treatment of ischemic stroke a single administration of azithromycin is expected to be sufficient to improve patient’s outcome, limiting the concerns associated with long-term antibacterial treatment. Notably, the evidence that administration of azithromycin in the acute phase enhances functional recovery in the chronic phase suggests that it may represent an effective pharmacological tool to reduce the burning of physical and behavioral disabilities, thus aiding rehabilitation programs for patients recovering from stroke.

In conclusion, our data demonstrate that azithromycin is neuroprotective against focal cerebral ischemia when administered as a single dose within a clinically relevant therapeutic window. Neuroprotection is associated with elevation of STAT3 phosphorylation in discrete cells populating the ischemic hemisphere, including neurons, astrocytes and M2-polarized myeloid cells that mediate the beneficial effects of the drug. Moreover, amelioration of histological and functional outcomes is long-lasting and is not associated with overt signs of toxicity. Thus, taken together, the results of the present work reaffirm the importance of developing azithromycin for the acute treatment of ischemic stroke.

## Data Availability Statement

The datasets generated for this study are available on request to the corresponding author.

## Ethics Statement

The animal study was reviewed and approved by the local committee (OPBA – University of Calabria) and by the Committee set by the Ministry of Health at the National Institute of Health (Rome) in agreement with the guidelines of the Italian Ministry of Health (DM 116/1992 and DL 26/2014) and the European Union Directive 2010/63/EU.

## Author Contributions

DA designed the study, wrote the manuscript, and contributed to acquisition, analysis, and interpretation of data. FP and RG contributed to data acquisition and analysis. CT, MC, PT, and GB supervised the work and edited the manuscript. All authors contributed to manuscript revision, and read and approved the submitted version.

## Conflict of Interest

The authors declare that the research was conducted in the absence of any commercial or financial relationships that could be construed as a potential conflict of interest.
